# Analysis of ADAM9 regulation and function in vestibular schwannoma primary cells

**DOI:** 10.1186/s13104-020-05378-7

**Published:** 2020-11-11

**Authors:** Anja Nattmann, Maria Breun, Camelia M. Monoranu, Cordula Matthies, Ralf-Ingo Ernestus, Mario Löhr, Carsten Hagemann

**Affiliations:** 1 Department of Neurosurgery, University Hospital Würzburg, Josef-Schneider-Str. 11, 97080 Würzburg, Germany; 2 Department of Neuropathology, Institute of Pathology, University of Würzburg, 97080 Würzburg, Germany

**Keywords:** Vestibular schwannoma, Pathogenesis, ADAM9, Knock down, Integrin, Immunofluorescence double staining, Merlin, Primary cell culture

## Abstract

**Objective:**

Recently, we described a disintegrin and metalloproteinase 9 (ADAM9) overexpression by Schwann cells of vestibular schwannoma (VS) and suggested that it might be a marker for VS tumor growth and invasiveness. This research note provides additional data utilizing a small cohort of VS primary cultures and tissue samples. We examined whether reconstitution of Merlin expression in VS cells regulates ADAM9 protein expression and performed lentiviral ADAM9 knock down to investigate possible effects on VS cells numbers. Moreover, the co-localization of ADAM9 and Integrins α6 and α2β1, respectively, was examined by immunofluorescence double staining.

**Results:**

ADAM9 expression was not regulated by Merlin in VS. However, ADAM9 knock down led to 58% reduction in cell numbers in VS primary cell cultures (p < 0.0001). While ADAM9 and Integrin α2β1 were co-localized in only 22% (2 of 9) of VS, ADAM9 and Integrin α6 were co-localized in 91% (10 of 11) of VS. Therefore, we provide first observations on possible regulatory functions of ADAM9 expression in VS.

## Introduction

Vestibular schwannoma (VS) are benign tumors emerging from Schwann cells of the vestibular part of the 8^th^ cranial nerve [1]. Their hallmark mutation is loss of Merlin function, which is a 4.1 protein/ezrin/radixin/moesin protein (FERM) and acts as a tumor suppressor protein inhibiting Schwann cell growth by connecting the cytoskeleton with the cell membrane. It is activated by the cells’ attachment to the extracellular matrix (ECM) and intercellular adhesion [2]. A disintegrin and metalloproteinase 9 (ADAM9) is involved in tumor growth and invasion by liberating membrane-bound proteins by an enzymatic modification called “shedding” [3]. Its proteolytic activity also releases cytokines and growth factors and it modifies the ECM by interacting with integrins [4]. In previous experiments we showed an 8.8 times higher ADAM9 mRNA-expression in VS compared to healthy vestibular nerves and suggested that ADAM9 inhibition may be of significance for VS pathogenesis and potential medical treatment [5]. Furthermore, we discussed whether ADAM9 might interact with the VS cell–matrix [5].

This research note builds on our recent findings. We hypothesized that alteration of Merlin expression could regulate ADAM9 protein expression and that ADAM9 plays a role in VS cell proliferation. Finally, we investigated co-localization of ADAM9 with Integrin α6 and Integrin α2β1, respectively, aiming to get a rough idea on putative regulatory protein interactions in VS.

## Material and methods

### Tissue samples and cell culture

Tissue of 24 VS, surgically resected in the Department of Neurosurgery of the University Hospital Würzburg, was collected from January 2018 until July 2019. Half of each sample was embedded in paraffin for immunohistochemistry, the other half was processed for primary cell culture as described elsewhere [6] and briefly outlined in Additional file 1.

### Merlin overexpression

95,000 cells per well of the primary cell cultures were treated with 2 ml VS-medium (see Additional file 1) containing 8 µg/ml protamine (Sigma, Munich, Germany) and 100 µl NF2 transcript variant 1 (NM_000268) Human mGFP Tagged ORF Clone Particle RC205883L2 (OriGene, Rockville, MD, USA) and incubated for 2 days. The medium was replaced by 2 ml fresh VS-medium, cells were photographically documented and lysed as described below.

### ADAM9 knock down

Cells were prepared as described above, but 57 µl ADAM9 Human shRNA Lentiviral Particle TL314947VB (Locus ID 8754) (knock down) or 120 µl Lenti-shRNA Control Particles TR30021V (scrambled control) (OriGene, Rockville, MD, USA) were added. After 3 days the medium was replaced by 2 ml VS-medium and 24 h later by 2 ml VS-medium containing 1 µg/ml puromycin (Gibco, Carlsbad, CA, USA), which was exchanged every 2 days by DMEM. At day 11 five fields of view of each well were photographed through the 10 × magnifying objective of the DMI 3000B fluorescence microscope and the DFC 450C camera (Leica, Wetzlar, Germany) in bright field with 35 ms exposure time and for green fluorescence using filter L5 ET and an exposure time of 900 ms. Cells were counted after importing the photographed fields of view to the program ImageJ by applying the tool “multi point” (National Institutes of Health (NIH), Bethesda, MD, USA; https://imagej.nih.gov/ij/).

### Western-blot

After washing the cells twice with phosphate buffered saline (PBS; Biochrom, Berlin, Germany), they were lysed with 100 µl lysis buffer [10 mM Tris–HCl pH 7.4, 150 mM NaCl, 1 mM ethylenediaminetetraacetic acid (EDTA), 1 mM ethylene glycerol-bis (β-aminoethylether)-N,N,N′,N′-tetraacetic acid (EGTA), 1% Triton X-100, 0.5% IGEPAL CA-630, 1 mM phenylmethanesulfonylfluoride (PMSF), 10 µg/ml leupeptin and 23 µg/ml aprotinin (all from Sigma, Munich, Germany)]. The extracted protein was measured with the Qubit 2.0 Fluorometer (Thermo Fisher Scientific, Waltham, MA, USA). 0.3 µg total protein was loaded to a polyacrylamid gel, electrophoresis was performed as described [7] and the gel blotted for 7 min using the iBlot system (Thermo Fisher Scientific, Waltham, MA, USA) set to program 3. The membrane was blocked in TBST (Sigma, Munich, Germany) containing 5% non-fat milk powder (Roth, Karlsruhe, Germany) and probed with antibodies as described previously [7]. Antibodies NF2 B-12 sc-55575 (Santa Cruz Biotechnology, Dallas, TX, USA), ADAM9 ab186833 (Abcam, Cambridge, UK), and anti-γ-tubulin T6557 (Sigma, Munich, Germany) were utilized diluted in TBST at 1:200, 1:1500, and 1:5000, respectively. Goat anti-rabbit IgG H&L (HRP) ab6721 (Abcam, Cambridge, UK), anti-mouse IgG HRP NA931 (GE Healthcare, Freiburg, Germany) and anti-mouse m-IgGk BP-HRP sc516102 (Santa Cruz Biotechnology, Dallas, TX, USA) antibodies were used as secondary antibodies at a 1:1000 dilution in TBST. The ECL Western Blotting Analysis System (Amersham, Freiburg, Germany) was used to visualize the antibody labeled proteins.

### Immunofluorescence double-staining

Immunofluorescence staining of 3 µm thick formalin-fixed paraffin sections has been described previously [7, 8]. However, blocking with 10% goat serum (Life Technologies, Waltham, MA, USA) was performed for 2 h prior to an incubation of the slides with antibody dilution buffer (DCS, Jena, Germany) containing antibodies anti-ADAM9 ab186833 (1:100) in combination with anti-Integrin α2β1 [16B4] ab30483 (1:50) or anti-Integrin α6 [MP 4F10] ab20142 (1:50) (all from Abcam, Cambridge, UK) at 4 °C overnight. Protein expression was visualized by 1 h incubation using Goat anti-Rabbit IgG (H + L) Highly Cross-Adsorbed Secondary Antibody (Alexa Fluor Plus 488 (A32732) and 555 (A32732); Thermo Fisher Scientific, Waltham, MA, USA), both diluted 1:400. Slides were mounted using Fluoroshield mounting medium, containing DAPI (Abcam, Cambridge, UK) and photographed with the Leica microscope DMI 3000B and the DFC 450C camera, using three different filters (Filtercubes A, L5 ET, and TXR ET) with exposure times of 77 ms, 2.5 s and 1.5 s, respectively, at both 10 and 40× objective magnification. The percentage of positively stained tumors in relation to all stained tumors was calculated.

### Statistical analysis

Statistical analysis was performed with GraphPad Prism 6 software (GraphPad Software, La Jolla, CA, USA) to determine significance using unpaired t tests. p < 0.05 was considered to be statistically significant.

## Results

**Fig. 1:**
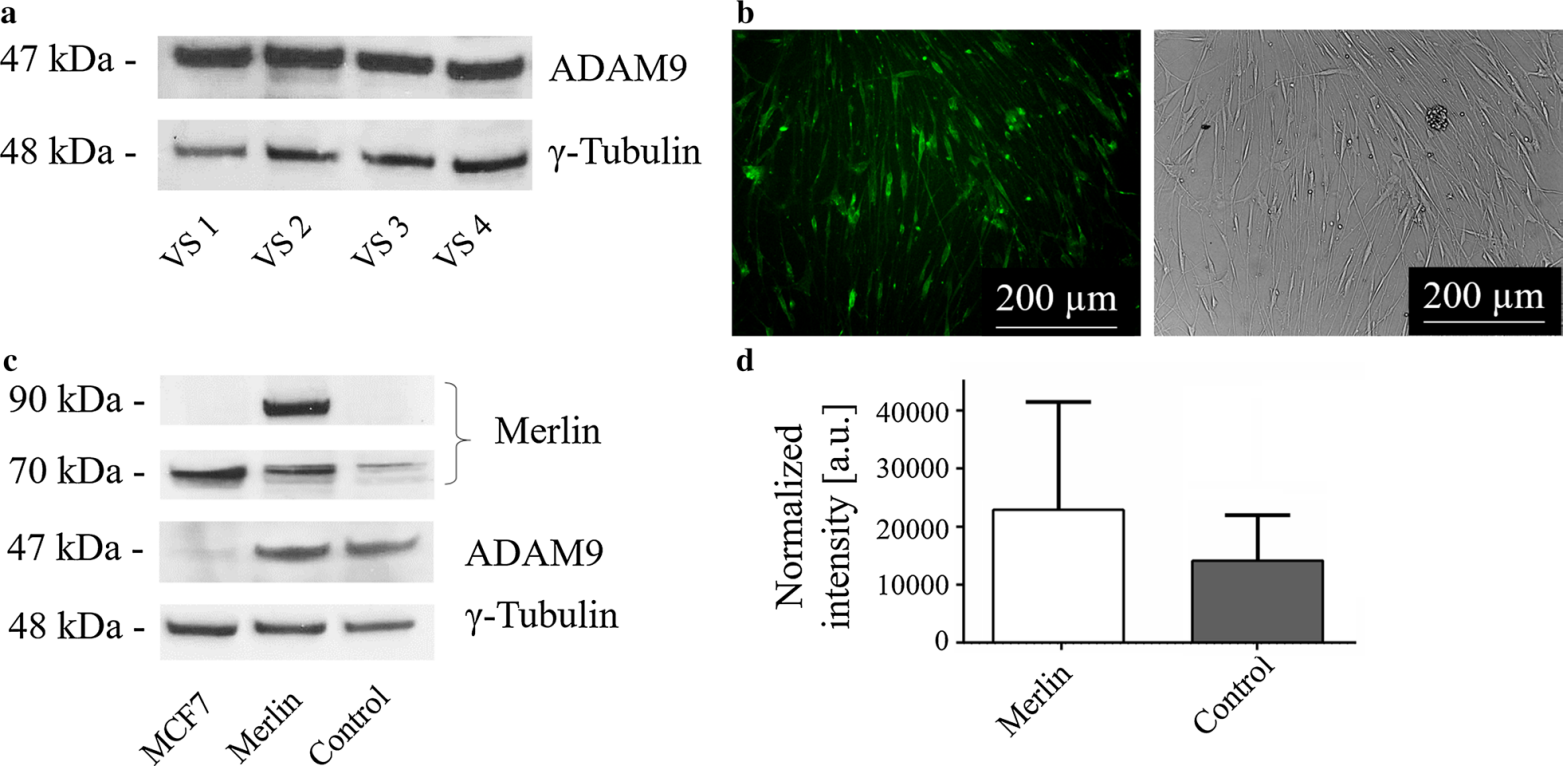
ADAM9 was not regulated by Merlin in VS. **a** Western-blot of ADAM9 expression in four representative VS of n = 24. **b** Merlin protein expression in VS primary cells after lentiviral transfection. The immunofluorescent images compare successfully transfected green fluorescent cells (left) with the same field of view showing all cells (right) to estimate the transfection rate. **c** Western-blot of MCF7 breast cancer cell-lysate (MCF7), suggested by the manufacturer of the Merlin antibody as positive control, VS primary cells transfected with Merlin (Merlin) and untransfected VS primary cells (Control). Cell lysates were loaded twice onto the same gel to avoid stripping of the blot, which then was cut into half for the incubation with Merlin and ADAM9 antibody, respectively. Shown is one representative experiment of n = 3. γ-Tubulin served as loading control in all Western-blot experiments. Blots were cropped for better clarity. The full length blots are presented in Additional file 1, Fig. S1 and full length blots of the two additional experiments in Additional file 1, Fig. S2a. **d** Quantification of ADAM9 expression in Merlin overexpressing (Merlin) and untransfected VS primary cells (Control) as analyzed in (**c**)

**Fig. 2:**
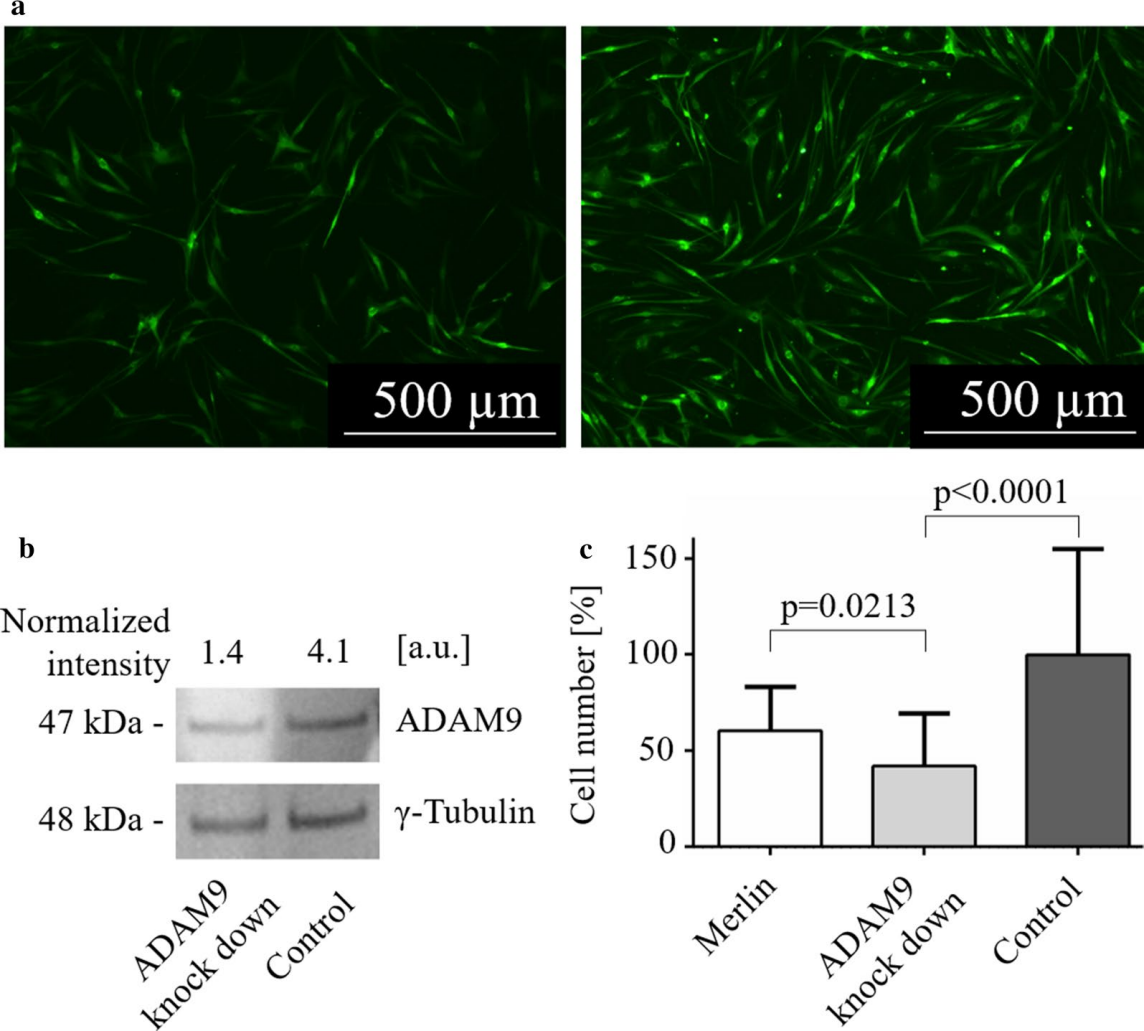
ADAM9 was involved in controlling VS primary cell proliferation. **a** Lentiviral mediated shRNA knock down of ADAM9. Green fluorescent ADAM9 knock down (left) and scrambled transfected control cells (right). One representative experiment of n = 4 is shown. **b** Proof of principle Western-blot of ADAM9 knock down, n = 1. γ-Tubulin served as loading control. Blots were cropped for better clarity. The full length blots are presented in Additional file 1, Fig. S2b. **c** Quantification of cell numbers after Merlin overexpression (Merlin) and ADAM9 knock down

Without exception, high amounts of ADAM9 were detectable in all 24 VS (Fig. 1a). Since Merlin loss is a hallmark of VS development [9], we wondered whether the ADAM9 expression was due to lacking regulation by Merlin. Therefore, we restored Merlin expression by lentiviral transfection (Fig. 1b) and checked for any alteration in ADAM9 expression utilizing Western-blots (Fig. 1c). ADAM9 expression levels, however, did not significantly differ in cells with or without Merlin expression (Fig. 1d), ruling out Merlin-mediated ADAM9 regulation. Next, we asked whether ADAM9 expression might be involved in the proliferation of VS cells. Therefore, a lentiviral shRNA-mediated ADAM9 knock down was performed (Fig. 2a). The ADAM9 shRNA led to a threefold reduction of ADAM9 compared to scrambled control (band intensity normalized to γ-Tubulin expression 1.4 vs. 4.1, respectively) in a proof of principle experiment (Fig. 2b). While Merlin overexpression did not result in statistically significant reduced cell numbers in comparison to vector-transfected controls, ADAM9 knock down caused a significant 58% reduction of VS cell numbers in comparison to the scrambled controls (p < 0.0001) and to 18% reduction in comparison to Merlin overexpressing primary cells (p = 0.0213) (Fig. 2c).

**Fig. 3:**
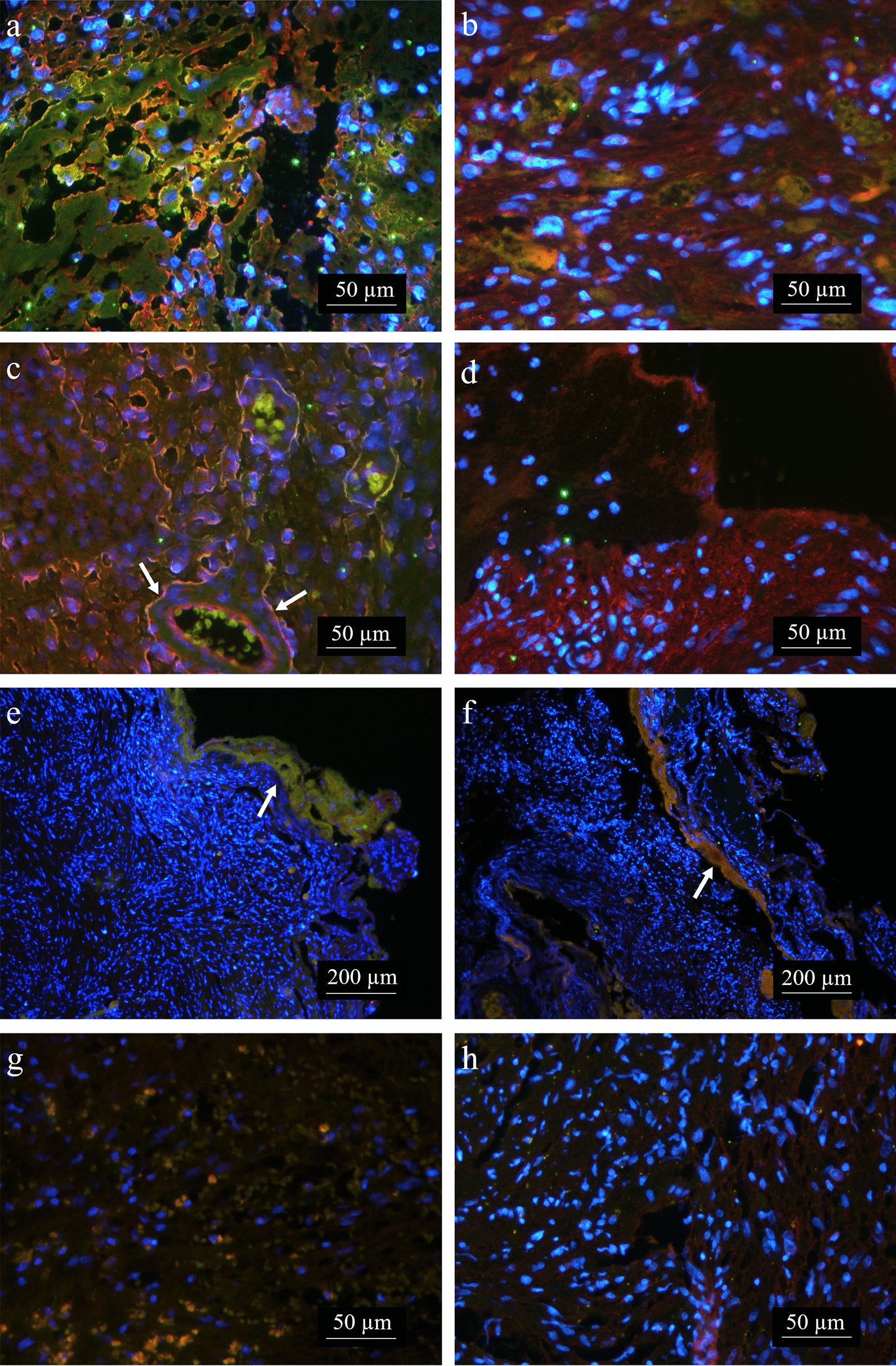
Co-localization of ADAM9 with Integrin α6 and Integrin α2β1 in VS tumor samples. **a**–**f** Immunofluorescence double-staining of ADAM9 (red) and Integrin α6 (green). **a**, **b** Co-localization (yellow) was mainly found in tumor tissue and **c** near blood vessels (arrows). **d** One VS did not express any detectable Integrin α6. **e**, **f** Demonstrate enriched co-localization along the tumor capsule (arrows). **g**, **h** Immunofluorescence double-staining of ADAM9 (red) and Integrin α2β1 (green). **g** Integrin α2β1 VS with ADAM9 co-localization. **h** Integrin α2β1 negative VS. DAPI = blue. Representative images of n = 20. The split-channel images are provided in Additional file 1 Fig. S3

ADAM9 is a cell–matrix modifying enzyme, reported to interact with Integrin α6β1 and thereby regulating cellular motility [10]. This integrin is also expressed by VS cells [10] and therefore could be associated with ADAM9 in VS. Indeed, Integrin α6 expression could be found in 10 of 11 investigated VS (91%) and was co-localized with ADAM9 (Fig. 3). This co-localization was mainly found in tumor tissue (Fig. 3a, b), near blood vessels (Fig. 3c) and enriched along the tumor capsule (Fig. 3e, f). In contrast, Integrin α2β1 was only weakly expressed in the analyzed VS (Fig. 3g, h). Strong expression with scattered ADAM9 co-localization was observed in merely two of 9 VS (22%) (Fig. 3g).

## Discussion

We were the first to describe ADAM9 overexpression in VS, suggesting it might be a marker for tumor growth and invasiveness [5]. However, our study was descriptive in nature and we only could speculate on ADAM9 regulation and function. Here, we performed first experiments to address these questions. We confirmed expression of ADAM9 in the analyzed VS, showed that Merlin overexpression does not regulate expression of the 47 kDa ADAM9 isoform, but that an ADAM9 knock down resulted in reduced VS cell numbers. Furthermore, we demonstrated ADAM9 co-localization with Integrin α6 in 91% of the analyzed VS, but with Integrin α2β1 only in 22% of the cases.

Merlin modulates cell–cell interactions and cell migration. It is a tumor suppressor gene, with loss of function mutations in VS [11], leading to overexpression of Neuregulin 1 (NDRG1), which in turn activates ERK- and AKT-signaling pathways in VS, promoting VS cell proliferation [12–14]. Since we observed overexpression of ADAM9 in VS, we wondered whether this may be due to the loss of Merlin in these cells and restored Merlin expression. However, our data suggest that there is no causal link between loss of Merlin and overexpression of at least the proteolytically-processed, membrane-bound 47 kDa isoform of ADAM9 [3]. However, the knock down of ADAM9 led to a reduction of VS cell numbers. ADAM9 inhibition had already been shown to reduce migration and invasion of human glioma cell lines [15]. Therefore, our data might be a first hint that ADAM9 plays a similar role in VS and its expression might induce progression or possibly reduce cell death in these benign tumors.

ADAM9 is a cell membrane spanning protein connecting the cytoskeleton with the ECM or neighboring cells. Its cysteine-rich domain binds to proteoglycans and its disintegrin domain to integrins [3]. Metalloproteinases of the ADAM-family digest proteins of the ECM, allowing tumor cells to detach from the tissue and thereby promoting their migration and invasion [3, 16] Thus, highest ADAM9 expression could be detected in areas of liver metastases with high invasive growth [3]. Integrins mediate attachment of cells to the basal lamina [17]. Integrin α6 and Integrin α2β1 are both known substrates of ADAM9. Interaction of ADAM9 and Integrin α2β1 has been described for liver metastases [3] and of ADAM9 and Integrin α6β1 for fibroblasts [10]. However, their association had not yet been shown for VS. Since we found co-localization of ADAM9 and Integrin α2β1 in only 22% of the analyzed VS, we conclude that this is not the main substrate of ADAM9 in VS. On the other hand, Integrin α6 is detectable in Schwann cells [18]. Its precursor has higher expression levels in VS cells in comparison to Schwann cells [19] and we found co-localization in 91% of the VS with highest levels in tumor tissue and near blood vessels. ADAM9 knock-out mice display a significant reduction of retinal neovascularization, whereas ADAM9 was highly expressed in pathological retinal blood vessels of wild type mice [20]. It has been suggested that hypoxia induces ADAM9 expression and this enhanced expression in turn leads to release of pro-angiogenic factors [20]. Our data may support such assumption and could indicate that VS may require neoangiogenesis for their growth, although they are generally considered to be weakly vascularized tumors [21].

Our new data supplement our previous observations on ADAM9 expression in VS, are a first glimpse on possible regulatory functions and provide a rationale for more in-depth future investigations.

## Limitations

VS are slowly growing, benign tumors. Thus, it is a challenge to establish primary cell cultures [6] and even more difficult to achieve high transfection rates. For this reason, only a small number of experiments could be performed for protein knock down and overexpression, limiting generalizability of these experiments. Several ADAM9 isoforms have been described [3, 22, 23]. However, the antibody used in this study was specific for the 47 kDa isoform only. Therefore, we cannot conclude on a possible Merlin mediated regulation of other ADAM9 isoforms. The immunofluorescence double staining indicates co-localization of ADAM9 and Integrin α6 or α2β1, which does not necessarily mean that there is direct protein–protein interaction.

## Supplementary information

**Additional file 1:** Additional details pertaining to material and methods. Details of cell culture. **Figure S1.** Unaltered full-length Western-blots of ADAM9, Merlin and γ-Tubulin protein expression in VS and VS primary cells from Fig. 1. **Figure S2.** Unaltered full-length Western-blots of Merlin, ADAM9 and γ-Tubulin protein expression in VS primary cells. **Figure S3.** Split-channel presentation of ADAM9 co-localization with Integrin α6 and Integrin α2β1 in VS tumor samples as shown in Fig. 3.

## Data Availability

The datasets generated and analyzed during the current study are included in this published article and its additional information files. Raw data are available from the corresponding author on reasonable request.

## Abbreviations

ADAM9: A disintegrin and metalloproteinase 9
DAPI: 4′,6-Diamidin-2-phenylindol
DMEM: Dulbeccos’s modified Eagle’s medium
ECM: Extracellular matrix
EDTA: Ethylenediaminetetraacetic acid
EGTA: Ethylene glycerol-bis (β-aminoethylether)-N,N,N′,N′-tetraacetic acid
FERM: 4.1 Protein/ezrin/radixin/moesin protein
IBMX: 3-Isobutyl-1-methylxanthin
NDRG1: Neuregulin 1
PBS: Phosphate buffered saline
PMSF: Phenylmethanesulfonylfluoride
TBST: Tris-buffered saline Tween 20
VS: Vestibular schwannoma
