# Supplementary material for: Analysis of ADAM9 regulation and function in vestibular schwannoma primary cells
Source: BMC Res Notes. 2020 Nov 11;13:528. doi: 10.1186/s13104-020-05378-7 (PMC7659081; doi:10.1186/s13104-020-05378-7)
Supplement: Supplementary file 1 — Additional file 1: Additional details pertaining to material and methods. Details of cell culture. Figure S1. Unaltered full-length Western-blots of ADAM9, Merlin and γ-Tubulin protein expression in VS and VS primary cells from Fig. 1. Figure S2. Unaltered full-length Western-blots of Merlin, ADAM9 and γ-Tubulin protein expression in VS primary cells. Figure S3. Split-channel presentation of ADAM9 co-localization with Integrin α6 and Integrin α2β1 in VS tumor samples as shown in Fig. 3. [file 13104_2020_5378_MOESM1_ESM.pdf]

Additional Materials

Analysis of ADAM9 regulation and function in vestibular schwannoma primary cells

Anja Nattmann, Maria Breun, Camelia M. Monoranu, Cordula Matthies, Ralf-Ingo Ernestus, Mario Löhr and Carsten Hagemann

Additional materials and methods

Cell culture

The fresh tumor samples were washed three times with ice-cold HBSS +/+ (Gibco, Carlsbad, CA, USA), shred into 0.2 × 0.2 cm small pieces and digested with 1.7 mg/ml dispase (PluriSTEM, Millipore, Massachusetts, USA) and 1.5 mg/ml collagenase (Sigma, Munich, Germany) in 6 ml VS-medium (DMEM supplemented with 10% heat-inactivated fetal bovine serum, 1% penicillin/streptomycin (all from Gibco, Carlsbad, CA, USA), 0.35% Glucose (B. Braun, Melsungen, Germany), 8 nM Heregulin (Stemcell Technologies, Cologne, Germany), 0.5 mM IBMX and 0.25 µg/ml insulin (both from Sigma, Munich, Germany)) for 17 h at 37°C and 5% CO_2. Afterwards, the suspension was centrifuged for 5 min at 300 ×g, the supernatant was discarded and the pellet resuspended in 12 ml VS-medium, before plating 2 ml into each well of laminated 6-well-plates [5]. The cells were cultured to 80% confluence in a humidified atmosphere of 5% CO_2 at 37°C.

Altogether, primary cell cultures of 24 VS were prepared. However, due to the growth limitations of VS primary cultures, as outlined in the limitations section of the main paper, not all experiments were performed with all 24 tumors. ADAM9 protein expression was determined by Western-blotting in all 24 VS and by immunofluorescence in 20 of them. Merlin overexpression was analyzed in a selection of 3, proof of ADAM9 knock down by Western-blot was evaluated in 1 and the ADAM9 knock down studies were performed utilizing 4 of the 24 VS primary cell cultures.

Additional figures

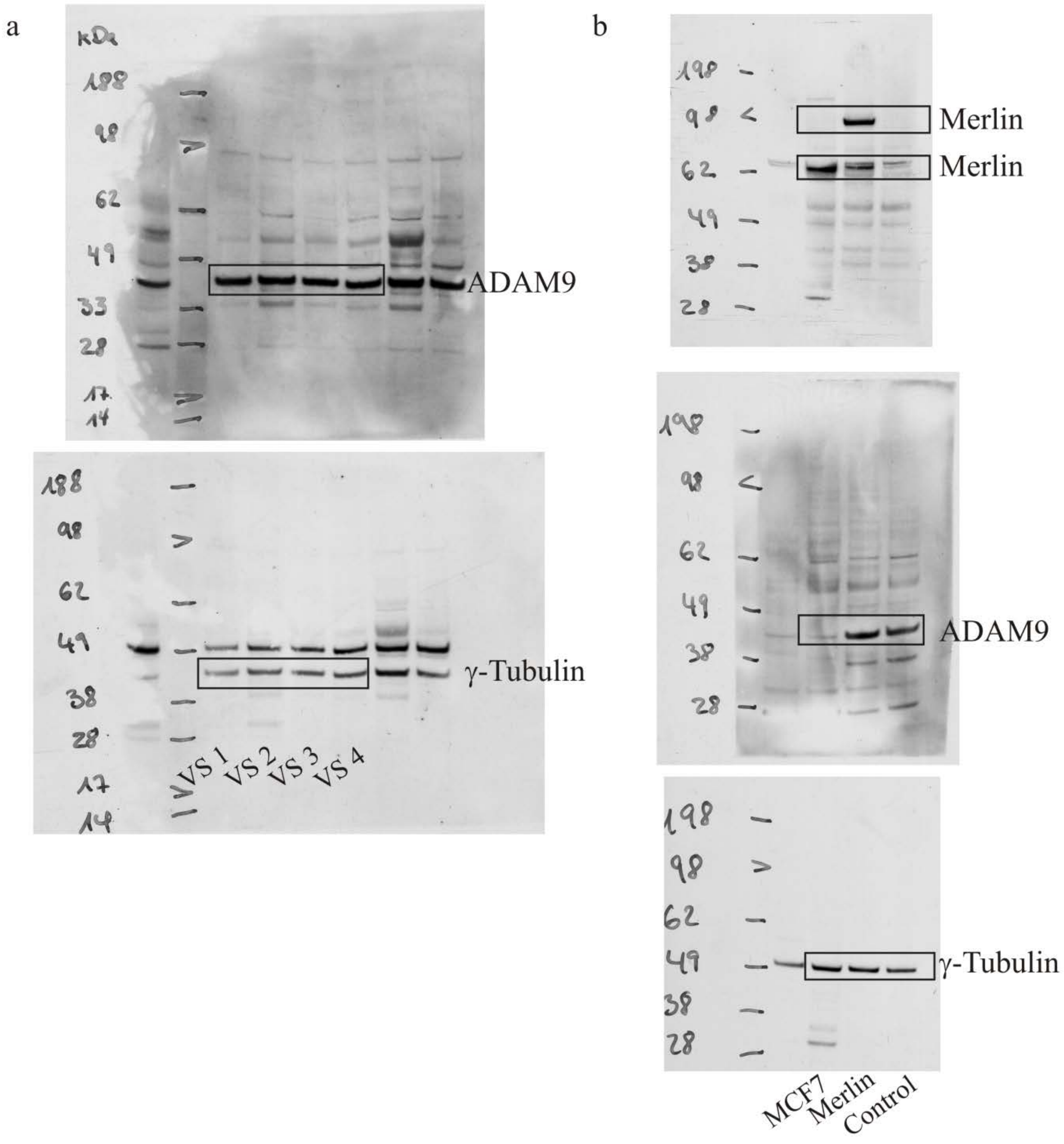

Figure S1. Unaltered full-length Western-blots of ADAM9, Merlin and γ-Tubulin protein expression in VS and VS primary cells from Figure 1. **a** Western-blot of ADAM9 expression in four representative VS. **b** Merlin and ADAM9 protein expression in VS primary cells after lentiviral Merlin transfection. Western-blot of MCF7 breast cancer cells (MCF), VS primary cells transfected with Merlin (Merlin) and untransfected VS primary cells (Control). Shown is one representative experiment of n = 3 (for the other 2 experiments, refer to Figure S2a). γ-Tubulin served as loading control in all Western-blot experiments. The black rectangles indicate the area where the blots were cropped for presentation in Figure 1.

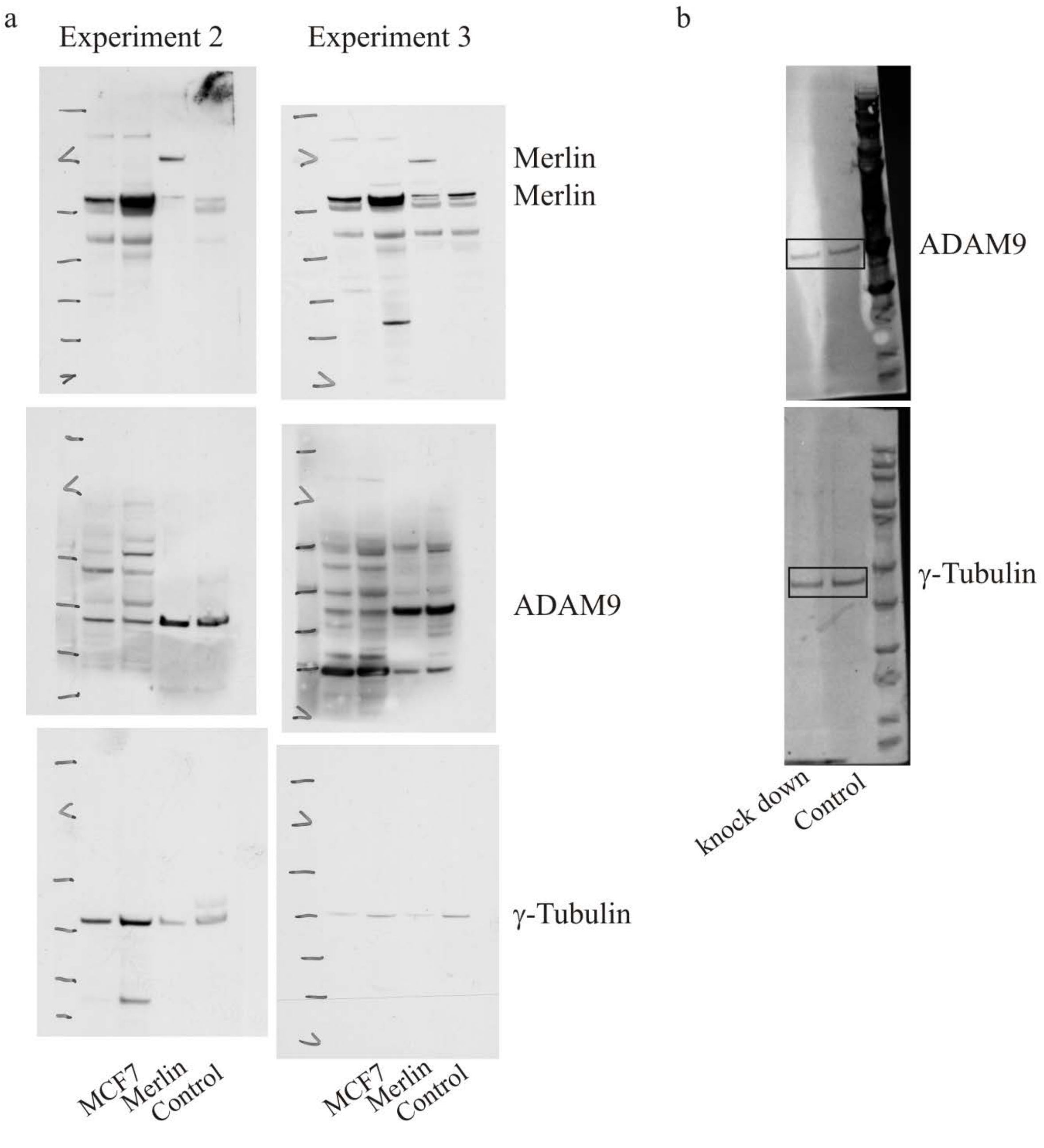

Figure S2. Unaltered full-length Western-blots of Merlin, ADAM9 and γ-Tubulin protein expression in VS primary cells. **a** Additional lentiviral Merlin transfection experiments in VS primary cells displaying Merlin and ADAM9 protein expression. Western-blot of MCF7 breast cancer cells (MCF), VS primary cells transfected with Merlin (Merlin) and untransfected VS primary cells (Control). **b** Proof of principle Western-blot of ADAM9 knock down in VS primary cells after lentiviral shRNA (knock down) and scrambled RNA (Control) transfection. n = 1. γ-Tubulin served as loading control in all Western-blot experiments. The black rectangles indicate the area where the blots were cropped for presentation in Figure 2b.

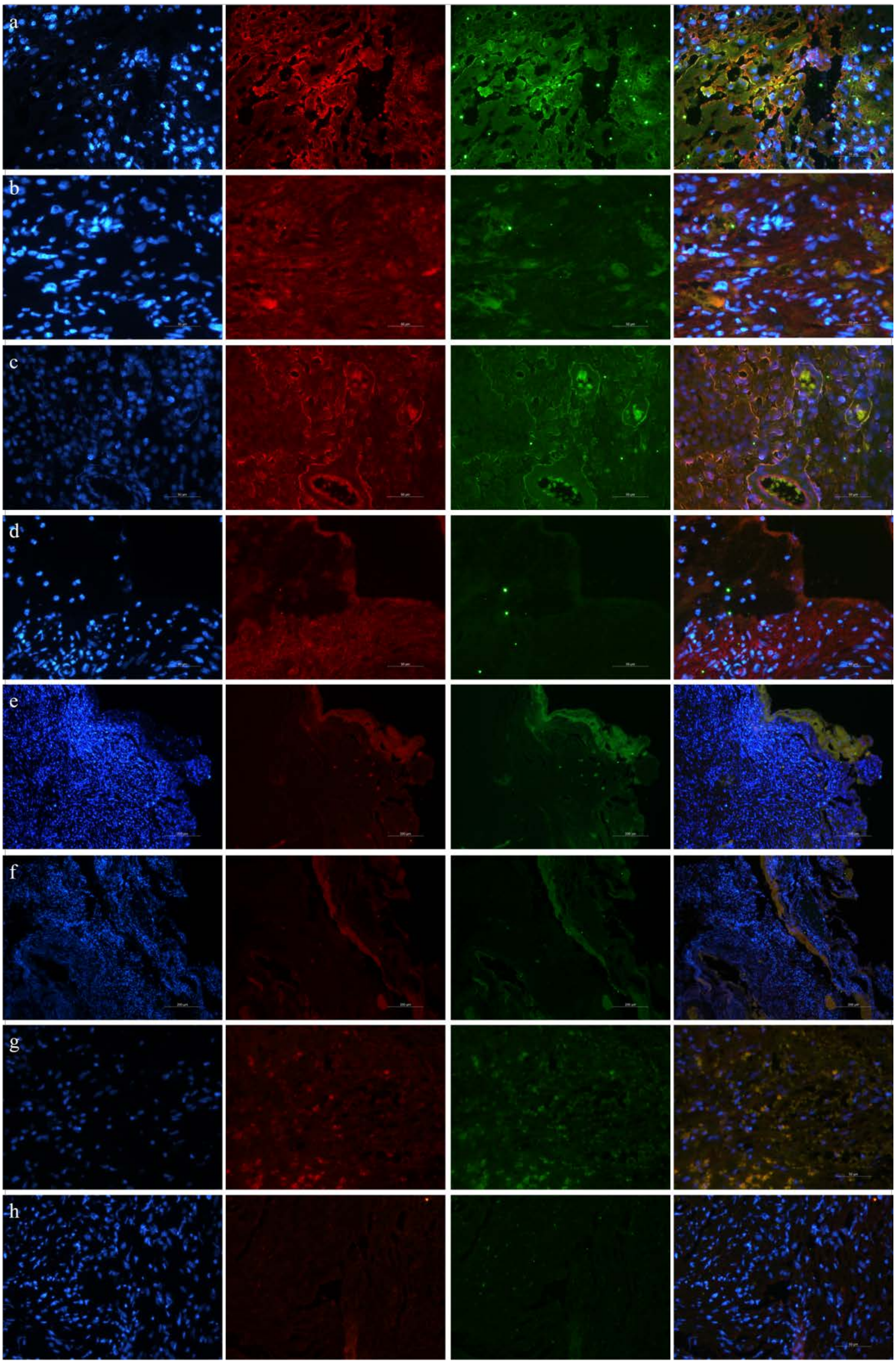

Figure S3. Split-channel presentation of ADAM9 co-localization with Integrin $\alpha6$ and Integrin $\alpha2\beta1$ in VS tumor samples as shown in Figure 3. **a-f** Immunofluorescence double-staining of ADAM9 (red) and Integrin $\alpha6$ (green) in tumor tissue (a, b), near blood vessels (c) and along the tumor capsule (e, f). The tumor shown in (d) was negative for Integrin $\alpha6$. **g-h** Immunofluorescence double-staining of ADAM9 (red) and Integrin $\alpha2\beta1$ (green). The tumor shown in (h) was negative for Integrin $\alpha2\beta1$. The outer left column shows DAPI staining of nuclei (blue), the outer right panel the merged images as they are shown in Figure 3 of the main manuscript.
